# Stepwise 900 µm to 1100 µm Microneedle Selection for Suprachoroidal Triamcinolone Acetonide

**DOI:** 10.1177/24741264261469695

**Published:** 2026-09-23

**Authors:** Victor Bellanda, Waseem Midlij, Danny A. Mammo

**Affiliations:** 1 Cole Eye Institute, Cleveland Clinic, Cleveland, OH, USA; 2 Ben-Gurion University of the Negev, Beersheba, Israel

**Keywords:** suprachoroidal injection, triamcinolone acetonide, microneedle selection, uveitic macular edema, scleral thickness, race, drug delivery

## Abstract

**Purpose:**

To describe real-world microneedle size use during suprachoroidal triamcinolone acetonide injection and identify patient-level factors associated with 900 µm vs 1100 µm microneedle requirement.

**Methods:**

A retrospective study was performed of all eyes receiving suprachoroidal triamcinolone acetonide between November 2022 and May 2026 from a single provider at a tertiary referral center. The primary outcome was microneedle size requirement (900 µm vs 1100 µm) at the first injection. The secondary outcome was needle size transition on repeat injections among eyes initially injected with the 900 µm needle. Generalized estimating equations with patient-level clustering were used for the primary analysis. Safety outcomes were also assessed.

**Results:**

A total of 103 eyes from 88 patients were included, comprising 202 injections. Using a standardized stepwise approach of attempting the 900 µm needle at first injection in all eyes, 64 eyes (62.1%) were successfully injected, while 39 eyes (37.9%) required the 1100 µm needle. On multivariable analysis, self-identified Black race was independently associated with requirement of a 1100 µm needle (adjusted odds ratio, 8.59, 95% CI, 2.42–30.48; *P* < .001), robust across primary, multivariable, and sensitivity analyses. No other demographic or ocular variable was significantly associated with needle size. No adverse safety events were observed.

**Conclusions:**

A stepwise approach beginning with the 900 µm needle is safe and effective for most patients and may optimize suprachoroidal drug delivery across a diverse patient population. Self-identified Black race is strongly and independently associated with requirement of a 1100 µm microneedle for suprachoroidal injection, potentially reflecting ancestry-related differences in scleral and suprachoroidal anatomy.

## Introduction

Uveitic macular edema (ME) is a visually significant complication of intraocular inflammation (IOI) and represents one of the leading causes of vision impairment in patients with noninfectious uveitis.^1^ Before the approval of suprachoroidal triamcinolone acetonide (Xipere, Bausch + Lomb), the management of uveitic ME relied on a range of periocular and intraocular corticosteroid therapies, including periocular triamcinolone acetonide, intravitreal (IVT) triamcinolone acetonide, and IVT implants including the dexamethasone and fluocinolone acetonide devices, each carrying risks of intraocular pressure elevation and cataract progression.^1^ In October 2021, Xipere received approval from the United States Food and Drug Administration for the treatment of cystoid macular edema (CME) associated with uveitis, becoming the first and only medication specifically approved for suprachoroidal administration.^2^

The suprachoroidal space represents a distinct and anatomically unique compartment between the sclera and the choroid, enabling targeted drug delivery to the posterior segment while minimizing exposure to anterior structures.^3^ Compared with IVT delivery, this route of administration offers high bioavailability to chorioretinal tissues and the potential for sustained drug release while reducing the risk of complications such as elevated intraocular pressure and cataract formation.^4^ Beyond corticosteroids, the suprachoroidal space has emerged as a promising platform for a growing pipeline of therapeutics, including gene therapy vectors, antivascular endothelial growth factor agents, and other small molecule suspensions, reflecting the broader significance of this delivery route in the future of posterior segment pharmacotherapy.^4–6^

Suprachoroidal drug delivery is performed using a specialized microinjector device equipped with a microneedle designed to traverse the sclera and deposit medication into the suprachoroidal space. The Xipere delivery system is available with 2 microneedle lengths: 900 µm and 1100 µm. The selection of needle length is clinically significant, as successful suprachoroidal drug delivery depends on the needle tip reaching the suprachoroidal space without penetrating into the choroid or vitreous cavity.^7^ Scleral thickness varies among individuals^8,9^ and may be influenced by factors including age,^10,11^ sex,^12–14^ ocular biometry,^15^ and previous ocular procedures, such as scleral buckling^16^ and trabeculectomy.^17^ In clinical practice, injections are typically first attempted with the 900 µm needle; in patients with thicker scleras, however, this needle may be insufficient to reach the suprachoroidal space, necessitating transition to the 1100 µm needle to achieve successful drug delivery. Conversely, routine use of the longer needle in patients with thinner scleras may risk inadvertent IVT drug deposition. As suprachoroidal injections become more widely adopted, retina specialists continue to refine their technique, and questions have emerged regarding which patient-specific factors influence microneedle length requirements.

The present study describes the use of 900 µm vs 1100 µm microneedles across initial and sequential injections and the patient-level factors associated with microneedle size requirements.

## Methods

This was a single-provider, single-center retrospective study conducted at the Cleveland Clinic Cole Eye Institute. All patients who received suprachoroidal triamcinolone acetonide administered by a single vitreoretina and uveitis specialist (D.M.) between November 2022 and May 2026 were included. The study adhered to the tenets of the Declaration of Helsinki. Informed consent was not required because it involved use of de-identified patient data.

### Injection Technique

All injections were performed by one of the study authors (D.M.), a single vitreoretina and uveitis specialist who completed training with a Bausch + Lomb representative using the standard kit provided to Xipere users before initiating suprachoroidal injections in clinical practice. D.M. had not participated in suprachoroidal injection clinical trials and had no previous experience with the technique before beginning its administration in routine patient care. All first injections are attempted with the 900 µm microneedle. Subconjunctival lidocaine, a lid speculum, and 5% povidone-iodine were used for all injections. Per the Xipere prescribing information, successful suprachoroidal drug deposition is indicated by loss of resistance as the plunger advances during injection; persistent resistance suggests the needle tip has not traversed the sclera to reach the suprachoroidal space.^18^ If the 900 µm needle was unsuccessful by this criterion, the 1100 µm needle was used for that same visit. If a patient required the 1100 µm needle at any injection, this was documented in the procedure note and electronic medical record and the 1100 µm needle used for all subsequent injections in that eye.

The superotemporal quadrant was the preferred injection site. When a glaucoma drainage device or trabeculectomy bleb precluded this site, the superonasal quadrant was selected. In rare cases where both superior quadrants were unavailable due to a broad bleb or significant Bell’s phenomenon, the inferotemporal quadrant was used. Injection site was not formally documented in the medical record; however, the superotemporal quadrant was used for the vast majority of injections per the treating physician’s standard practice.

### Data Collection

The following variables were extracted for each eye: date of injection, patient age at first injection, self-reported race/ethnicity, phakic status, CME etiology (cataract surgery, other ocular surgery, or uveitis/inflammation without surgical trigger), prior ocular surgery (retinal, glaucoma, or corneal), presence of scleral buckle, history of glaucoma, diabetic retinopathy, high myopia, or retinal detachment, and microneedle size used at each injection (900 µm or 1100 µm). For patients receiving more than 1 injection, needle size was recorded for all sequential injections. Because some patients received bilateral injections, the unit of analysis was the eye rather than the patient.

### Statistical Analysis

The primary outcome was the microneedle size requirement (900 µm vs 1100 µm) at the first date of suprachoroidal injection. The secondary outcome was needle size transition among eyes that received the 900 µm needle at the first injection and had at least 1 subsequent injection on a different date, defined as switching from 900 µm to 1100 µm at any later injection. Safety outcomes included rates of endophthalmitis, suprachoroidal hemorrhage, inadvertent IVT triamcinolone acetonide deposition, IOI, retinal tear or detachment, and need for subsequent glaucoma surgery.

To account for the correlation of eyes within patients in bilateral cases, all regression models used generalized estimating equations with a logit link and patient-level clustering, with robust sandwich standard errors. For the primary outcome, univariable generalized estimating equation models were fit for each candidate variable. Categorical variables with more than 2 levels (race/ethnicity, CME etiology) were entered as dummy variables relative to a reference category, with an overall Wald test reported. Continuous variables were entered as-is, with odds ratios (OR) reported per 1-unit increase. Variables with a univariable *P* value less than .2 were carried forward into a multivariable generalized estimating equation model. Given very small cell sizes for Hispanic (n = 3) and Asian (n = 3) patients, multivariable models were restricted to comparing Black and White race.

Given the small sample size (9 switchers vs 21 nonswitchers), nonclustered tests were used for the secondary outcome, treating each eye as an independent observation. For binary variables, Fisher exact test was used when any expected cell count was less than 5; otherwise, Pearson χ^2^ test was used. For continuous variables, normality was assessed using the Shapiro-Wilk test; Welch’s *t* test was used when both groups passed normality and the Mann-Whitney *U* test otherwise. No multivariable model was fit for the secondary analysis due to the sample size. Results of the secondary analysis are considered hypothesis-generating only.

A sensitivity analysis was performed using an alternative outcome definition, considered as “ever received 1100 µm” vs “never received 1100 µm” across all injections, which reclassified the 9 eyes that started on 900 µm but subsequently switched to 1100 µm into the 1100 µm group. The rationale was that eventual need for the longer needle may reflect underlying scleral anatomy regardless of initial needle success. The same generalized estimating equation framework and multivariable variable selection approach used in the primary analysis were applied.

All analyses were performed using Python 3.11. A 2-sided *P* value < .05 was considered statistically significant.

## Results

A total of 103 eyes from 88 patients, comprising 202 injections, were included in the study. All eyes were initially approached with the 900 µm microneedle. Of these, 64 eyes (62.1%) from 57 patients were successfully injected with the 900 µm microneedle at their first injection, while the remaining 39 eyes (37.9%) from 33 patients required transition to the 1100 µm microneedle. The most common indication for suprachoroidal triamcinolone was persistent CME after phacoemulsification (34 eyes [53.1%] in the 900 µm group; 18 eyes [46.2%] in the 1100 µm group). Most eyes in both groups were pseudophakic (92.2% and 87.2%, respectively) (Table 1).

**Table 1.** Baseline Characteristics by Microneedle Size at First Injection.

Variable	900 µm (64 eyes, 57 patients)	1100 µm (39 eyes, 33 patients)
Mean age (y) ± SD	71.8 ± 9.5	70.8 ± 9.9
Female sex, n (%)	31 (48.4)	20 (51.3)
Race/Ethnicity, n (%)		
White	57 (89.1)	22 (56.4)
Black	4 (6.2)	14 (35.9)
Hispanic	2 (3.1)	1 (2.6)
Asian	1 (1.6)	2 (5.1)
CME etiology, n (%)		
Phacoemulsification	34 (53.1)	18 (46.2)
Other ocular surgery	16 (25.0)	10 (25.6)
Uveitis/Inflammation without surgical trigger	14 (21.9)	11 (28.2)
CME duration (d), median (IQR)	11.8 (3.9-34.0)	13.0 (4.7-23.9)
Pseudophakia, n (%)	59 (92.2)	34 (87.2)
Prior ocular procedure other than phacoemulsification, n (%)	28 (43.8)	13 (33.3)
Prior scleral buckle, n (%)	5 (7.8)	4 (10.3)
Prior cornea surgery, n (%)	5 (7.8)	1 (2.6)
Prior glaucoma procedure, n (%)	11 (17.2)	4 (10.3)
Prior retina surgery, n (%)	14 (21.9)	10 (25.6)
Glaucoma, n (%)	24 (37.5)	14 (35.9)
High myopia, n (%)	4 (6.2)	1 (2.6)
Diabetic retinopathy, n (%)	6 (9.4)	3 (7.7)
History of retinal detachment, n (%)	6 (9.4)	6 (15.4)

Abbreviations: CME, cystoid macular edema; IQR, interquartile range.

### Primary Outcome: Microneedle Size at First Injection

**Figure 1.**
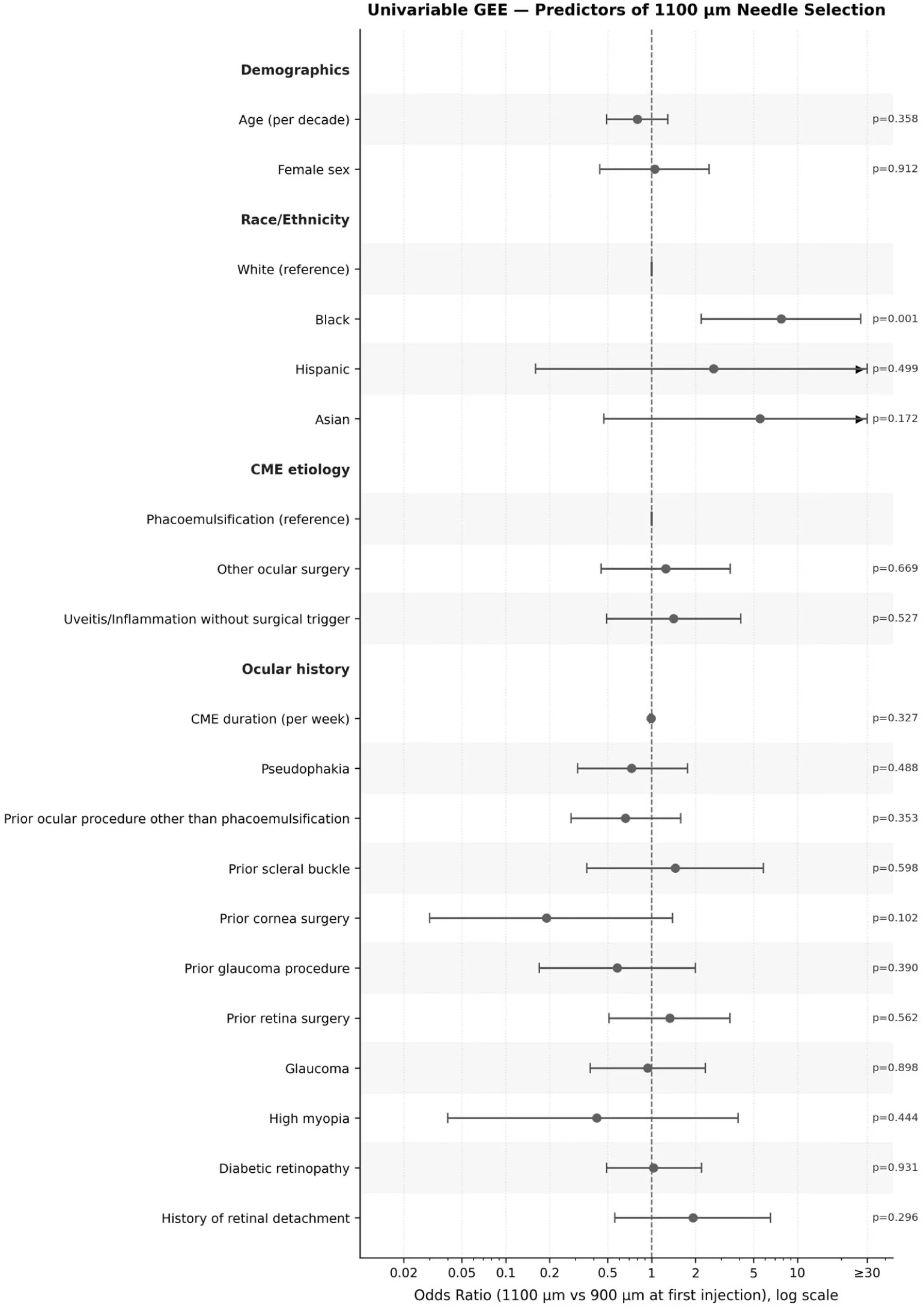
Univariable generalized estimating equation (GEE) forest plot showing odds ratios for 900 µm vs 1100 µm microneedle use at the first suprachoroidal injection. Each point represents the odds ratio estimate with 95% CIs displayed on a log scale. The dashed vertical line indicates an odds ratio of 1.0 (no association). White race, phacoemulsification, and absence of prior ocular procedures other than phacoemulsification served as reference categories for their respective variable groups. *P* values are displayed to the right of each confidence interval. Race/ethnicity was the only variable group with a statistically significant overall association with 1100 µm needle use (overall Wald; *P* = .010), driven by the comparison of Black and White race (odds ratio, 7.71, 95% CI, 2.19–27.17; *P* = .001). Hispanic and Asian patients showed point estimates in the same direction as Black patients, but this did not reach statistical significance, likely reflecting small group sizes (n = 3 each). No other demographic, ocular history, or comorbidity variable was significantly associated with microneedle size requirement at the first injection. Abbreviation: CME, cystoid macular edema.

In univariable generalized estimating equation analysis, self-reported race/ethnicity was the only variable significantly associated with 1100 µm needle use at the first injection (overall Wald; *P* = .010). Black race was associated with substantially higher odds of requiring the 1100 µm needle compared with White race (OR, 7.71, 95% CI, 2.19–27.17; *P* = .001). Univariable ORs for patients who identified as Hispanic or Asian trended in the same direction (ORs, 2.65 and 5.53, respectively) but did not reach statistical significance, likely reflecting the small number of patients in these groups (n = 3 each). No other variable, including age, sex, lens status, prior ocular surgery, glaucoma, scleral buckle, diabetic retinopathy, high myopia, or retinal detachment, was significantly associated with needle size at the first injection (Figure 1).

On univariable analysis, self-reported race/ethnicity (overall Wald; *P* = .010) and prior cornea surgery (*P* = .102) met the prespecified threshold of *P* < .2 for inclusion in the multivariable model; no other variables, including presence of scleral buckle or history of glaucoma procedures, met this threshold. In the multivariable generalized estimating equation model (restricted to Black vs White given small cell sizes for other race/ethnicity groups), Black race remained strongly and independently associated with use of a 1100 µm needle after adjusting for prior cornea surgery (adjusted OR, 8.59, 95% CI, 2.42–30.48; *P* < .001). Prior cornea surgery was not independently significant in this model (adjusted OR, 0.18, 95% CI, 0.01–3.77; *P* = .269). In a prespecified confirmatory model restricted to Black vs White race and age, the association with Black race was robust (adjusted OR, 8.25, 95% CI, 2.20–30.91; *P* = .002), and age was still not independently associated with needle size (adjusted OR, 1.01, 95% CI, 0.96–1.06; *P* = .722).

Among the 15 patients who received bilateral injections, needle size requirement was concordant between fellow eyes in 13 (86.7%), with 7 pairs requiring 900 µm and 6 pairs requiring 1100 µm in both eyes. Only 2 discordant pairs were observed (Cohen’s kappa = 0.737), suggesting substantial within-patient agreement in needle size requirement.

### Secondary Outcome: Needle Size Transition on Repeat Injections

Of the 64 eyes that received the 900 µm needle at their first injection, 30 eyes had at least 1 subsequent injection and were eligible for the secondary analysis. Of these, 9 eyes (30.0%) switched from 900 µm to 1100 µm at a later injection, while 21 (70.0%) continued receiving the 900 µm needle throughout all subsequent injections.

Given the small sample size, the secondary analysis was hypothesis-generating only. On nonclustered testing, female sex was more common among switchers than nonswitchers (66.7% vs 23.8%, Fisher exact; *P* = .042), and prior glaucoma surgery was more frequent in switchers (44.4% vs 9.5%, Fisher exact; *P* = .049). No other variable reached statistical significance. Age, CME etiology, self-reported race/ethnicity, lens status, prior retinal surgery, scleral buckle, and other ocular comorbidities did not differ between groups (Table 2).

**Table 2.** Baseline Characteristics of Eyes by Needle Size Transition Status on Repeat Injection.

Variable	Continued 900 µm (21 eyes, 19 patients)	Switched to 1100 µm (9 eyes, 9 patients)
Mean age (y) ± SD	69.9 ± 10.9	71.0 ± 7.4
Female sex, n (%)	5 (23.8)	6 (66.7)
Race/Ethnicity, n (%)		
White	18 (85.7)	8 (88.9)
Black	1 (4.8)	1 (11.1)
Hispanic	2 (9.5)	0 (0.0)
Asian	0 (0.0)	0 (0.0)
CME etiology, n (%)		
Phacoemulsification	10 (47.6)	4 (44.4)
Other ocular surgery	6 (28.6)	3 (33.3)
Uveitis/Inflammation without surgical trigger	5 (23.8)	2 (22.2)
CME duration (d), median (IQR)	14.6 (3.9-34.0)	11.7 (7.0-339.3)
Pseudophakia, n (%)	20 (95.2)	8 (88.9)
Prior ocular procedure other than phacoemulsification, n (%)	8 (38.1)	6 (66.7)
Prior scleral buckle, n (%)	2 (9.5)	1 (11.1)
Prior cornea surgery, n (%)	3 (14.3)	0 (0.0)
Prior glaucoma procedure, n (%)	2 (9.5)	4 (44.4)
Prior retina surgery, n (%)	4 (19.0)	2 (22.2)
Glaucoma, n (%)	9 (42.9)	4 (44.4)
High myopia, n (%)	3 (14.3)	0 (0.0)
Diabetic retinopathy, n (%)	2 (9.5)	0 (0.0)
History of retinal detachment, n (%)	2 (9.5)	1 (11.1)

Abbreviations: CME, cystoid macular edema; IQR, interquartile range.

### Sensitivity Analysis

When the outcome was redefined as ever having received the 1100 µm needle across any injection (48 eyes [46.6%]), the association with self-reported Black race was consistent with the primary analysis in both univariable (OR, 6.73, 95% CI, 1.74–26.06; *P* = .006) and multivariable models (adjusted OR, 7.89, 95% CI, 2.04–30.52; *P* = .003, adjusted for prior cornea surgery). Prior cornea surgery showed a trend toward lower odds of ever receiving the 1100 µm needle (adjusted OR, 0.12, 95% CI, 0.01–2.38; *P* = .165) but did not reach significance.

### Safety

No cases of endophthalmitis, suprachoroidal hemorrhage, inadvertent IVT triamcinolone acetonide deposition, IOI, retinal tear or detachment, or need for subsequent glaucoma surgery were observed.

## Conclusions

In our single-center experience with suprachoroidal triamcinolone acetonide, 62% of eyes were successfully injected with the 900 µm needle at their first attempt, and no cases of endophthalmitis, suprachoroidal hemorrhage, or IOI were observed across the entire series. The only patient-level factor independently associated with requiring the 1100 µm microneedle at the first injection was self-identification as Black, with an approximately 8-fold higher odds compared with patients who identified as White. This association was robust across univariable, multivariable, and sensitivity analyses.

In the absence of robust real-world data on microneedle selection, practitioners early in the learning curve may be inclined to default to the 1100 µm needle as a precaution against failed suprachoroidal injection. However, selection of needle length carries consequences in both directions: while too short a needle risks failing to reach the suprachoroidal space in patients with thick scleras, too long a needle risks traversing the suprachoroidal space entirely in patients with thin scleras, with potential for inadvertent intravitreal or intraocular drug deposition. Although published reports of such misdirection specifically from suprachoroidal injection are lacking, inadvertent intraocular corticosteroid deposition from periocular routes is a well-described phenomenon.^19,20^ The safety profile observed in this series, achieved through a standardized stepwise approach of attempting the 900 µm needle first in all patients, suggests that beginning with the shorter needle while reserving the 1100 µm needle for those in whom initial suprachoroidal deposition cannot be confirmed is not only safe but also effective for most patients. Tips for successful injection include holding the needle perpendicular to the scleral surface, gentle rocking back and forth with the fingertips while finding the suprachoroidal space, and rotating the needle 90 degrees while repeating these maneuvers. If these measures fail, then the 1100 µm needle would be used. All patients in this series received subconjunctival lidocaine with a minimum 10-minute waiting period before injection, although the numbing technique may vary by physician preference.

The strong and independent association between self-reported Black race and 1100 µm needle requirement observed in this study is perhaps explained by differences in scleral anatomy. The suprachoroidal space represents a potential space at the interface between the choroid and sclera, and its visibility and measurable thickness on enhanced-depth imaging optical coherence tomography (OCT) vary significantly by race. In a large cross-sectional study of healthy eyes, Chang et al^21^ found that both presence of suprachoroidal space on examination and subfoveal suprachoroidal space thickness were independently associated with White race, while other racial groups had substantially lower rates of suprachoroidal space detection even after adjusting for choroidal thickness and other confounders, suggesting a more compact or less patent choroidal-scleral interface in these groups, which would require greater needle depth to access the suprachoroidal space.

Ancestry-related differences in scleral properties and microarchitecture have also been documented. Grytz et al^22^ demonstrated that the posterior sclera in donors of African descent exhibits a significantly higher shear modulus and lower collagen fibril crimp angle, indicating a stiffer, less compliant tissue with altered biomechanical behavior compared with donors of European descent. Microstructural analysis of posterior scleral collagen architecture has similarly demonstrated racial differences, with African American donors showing a higher proportion of meridianally oriented fibers compared with Caucasian donors, a pattern that may further alter the mechanical and anatomic properties of the tissue at the injection site.^23^ Anterior scleral thickness itself shows significant regional and individual variability, with studies using anterior segment-OCT reporting substantial interindividual differences across meridians and demographic subgroups.^10,12^ Although our study did not directly measure scleral thickness or suprachoroidal space dimensions, the convergence of these anatomic and biomechanical findings across multiple modalities provides a mechanistic hypothesis for interpreting our clinical observation. The high concordance in needle size between fellow eyes (86.7%, kappa = 0.737) further supports a systemic anatomic basis for needle size requirement.

The secondary analysis, while hypothesis-generating given the small sample size, identified 2 potential factors associated with needle size transition: female sex and prior glaucoma surgery. The association with female sex is difficult to interpret in the context of the broader study findings. Sex was not associated with needle size requirement at the first injection in the primary analysis, and no clear directional hypothesis emerges from the literature, where sex-related differences in anterior scleral thickness, while reported, are modest and inconsistent across studies.^10,12^ We believe this association most likely reflects a chance finding given the very small number of switchers (n = 9). The association with prior glaucoma surgery, although equally uncertain, is more mechanistically interesting. Glaucoma procedures such as trabeculectomy and tube shunt implantation induce a robust wound healing response in the scleral and episcleral tissues, characterized by progressive fibrosis, collagen deposition, and structural remodeling.^24,25^ If this remodeling alters the mechanical properties of tissue near the injection site, it is conceivable that eyes that initially permitted suprachoroidal access with the shorter needle could develop increased tissue resistance over time, necessitating the longer needle at subsequent visits. In addition, when both superior quadrants were precluded by a broad bleb, injection was performed in the inferotemporal quadrant, where scleral thickness is greater.^11^ This could mechanistically contribute to the observed association between prior glaucoma surgery and needle size transition in a subset of eyes. These hypotheses, however, are speculative and require confirmation in larger series accounting for the timing of glaucoma procedures in relation to the needle switch. Both findings from this secondary analysis should be interpreted with caution.

This study has several important limitations. First, it is a single-center, single-provider retrospective series, and the findings may not be generalizable to other practices, patient populations, or injectors with different levels of experience or technique. Second, and most critically, neither the scleral thickness nor the dimensions of the suprachoroidal space were directly measured in any patient. Our interpretation of the race association as anatomically mediated is biologically plausible and supported by the existing literature but remains inferential. We cannot confirm that racial differences in scleral or suprachoroidal anatomy drove needle size requirements in our cohort, and future studies incorporating preprocedure anterior segment-OCT thickness measurement would be better positioned to test this hypothesis directly. Corneal pachymetry has been explored as a potential surrogate; however, multiple studies have found no significant correlation between central corneal thickness and anterior scleral thickness in normal eyes or primary open-angle glaucoma, with a weak correlation reported only in normal-tension glaucoma subgroups,^26,27^ further underscoring that direct scleral imaging would be necessary for anatomy-guided needle selection.

Beyond these methodological limitations, the use of race as a variable in this analysis warrants explicit acknowledgment. Race is a social and political construct, rather than a biological one, and its use as a proxy for underlying ancestry-related anatomic differences is imperfect and potentially misleading.^28,29^ The racial categories used in this study reflect self-reported designations registered on the electronic health records and aggregate substantial within-group heterogeneity. The association between Black race and 1100 µm needle requirement observed here likely reflects population-level differences in ocular anatomy that might correlate with ancestry, but race itself is not the causal variable; rather, it serves as an imprecise marker for a complex set of biological and possibly environmental factors that influence scleral and suprachoroidal anatomy.

The safety profile observed in this series is consistent with the established safety record of suprachoroidal triamcinolone acetonide across its pivotal trials. Across the phase 2 DOGWOOD trial, the phase 3 PEACHTREE trial, the open-label AZALEA safety study, and the MAGNOLIA extension trial, no cases of endophthalmitis, suprachoroidal hemorrhage, or treatment-related IOI were reported in any study eye receiving suprachoroidal injection.^30–33^ Postapproval real-world studies have similarly not reported these complications.^34,35^ The absence of these outcomes across all 202 injections in our series further supports the safety of suprachoroidal triamcinolone acetonide when administered using a careful, stepwise injection technique.

In conclusion, this single-center retrospective study demonstrated that a standardized stepwise approach of attempting the 900 µm needle first in all patients was safe and effective, with 62% of eyes successfully injected at their first attempt. Within this approach, self-identification as Black was the only patient-level factor independently and robustly associated with requirement of a 1100 µm microneedle at the first injection, with an approximately 8-fold higher odds compared with patients who self-identified as White. As suprachoroidal injection use expands across an increasingly broad range of indications, prospective studies incorporating direct scleral thickness measurement and suprachoroidal space imaging may help move beyond race-based inference and toward individualized, anatomy-guided needle selection.
